# Mitigating the impact of flip angle and orientation dependence in single compartment R2* estimates via 2‐pool modeling

**DOI:** 10.1002/mrm.29428

**Published:** 2022-09-26

**Authors:** Giorgia Milotta, Nadège Corbin, Christian Lambert, Antoine Lutti, Siawoosh Mohammadi, Martina F. Callaghan

**Affiliations:** ^1^ Wellcome Centre for Human Neuroimaging, UCL Queen Square Institute of Neurology University College London London United Kingdom; ^2^ Centre de Résonance Magnétique des Systèmes Biologiques, UMR5536 CNRS/University Bordeaux Bordeaux France; ^3^ Laboratory for Research in Neuroimaging, Department for Clinical Neuroscience Lausanne University Hospital and University of Lausanne Lausanne Switzerland; ^4^ Department of Systems Neurosciences University Medical Center Hamburg‐Eppendorf Hamburg Germany; ^5^ Department of Neurophysics Max Planck Institute for Human Cognitive and Brain Sciences Leipzig Germany

**Keywords:** mono‐exponential, multi‐compartment, R_2_* mapping, single compartment, T_2_*, VFA

## Abstract

**Purpose:**

The effective transverse relaxation rate ($R_{2}^{*}$) is influenced by biological features that make it a useful means of probing brain microstructure. However, confounding factors such as dependence on flip angle (α) and fiber orientation with respect to the main field (θ) complicate interpretation. The α‐ and θ‐dependence stem from the existence of multiple sub‐voxel micro‐environments (e.g., myelin and non‐myelin water compartments). Ordinarily, it is challenging to quantify these sub‐compartments; therefore, neuroscientific studies commonly make the simplifying assumption of a mono‐exponential decay obtaining a single $R_{2}^{*}$ estimate per voxel. In this work, we investigated how the multi‐compartment nature of tissue microstructure affects single compartment $R_{2}^{*}$ estimates.

**Methods:**

We used 2‐pool (myelin and non‐myelin water) simulations to characterize the bias in single compartment $R_{2}^{*}$ estimates. Based on our numeric observations, we introduced a linear model that partitions $R_{2}^{*}$ into α‐dependent and α‐independent components and validated this in vivo at 7T. We investigated the dependence of both components on the sub‐compartment properties and assessed their robustness, orientation dependence, and reproducibility empirically.

**Results:**

$R_{2}^{*}$ increased with myelin water fraction and residency time leading to a linear dependence on α. We observed excellent agreement between our numeric and empirical results. Furthermore, the α‐independent component of the proposed linear model was robust to the choice of α and reduced dependence on fiber orientation, although it suffered from marginally higher noise sensitivity.

**Conclusion:**

We have demonstrated and validated a simple approach that mitigates flip angle and orientation biases in single‐compartment $R_{2}^{*}$ estimates.

## 1 INTRODUCTION

Quantitative relaxometry offers great potential for characterizing brain microstructure.^1^, ^2^ The relaxation rates of water protons depend on the physical and chemical composition of the tissue,^3^ as well as on the rate at which the water molecules move between different micro‐environments^4^ making relaxometry sensitive to tissue microstructure on multiple spatiotemporal scales. For example, the effective transverse relaxation rate ($R_{2}^{*}$) is influenced by biologically‐relevant features such as iron content^5^ and myelination,^5^, ^6^ facilitating in vivo investigation of age‐related differences^7^ as well as pathological change.^8^, ^9^, ^10^, ^11^, ^12^

Neuroscientific studies commonly make the simplifying assumption of a mono‐exponential decay to obtain a single $R_{2}^{*}$ estimate per voxel. In reality the underlying microstructure is comprised of multiple distinct compartments. However, this simplification offers robustness to measurement noise, particularly when it is difficult to distinguish between the different compartments owing to rapid exchange between them, low SNR, or limited sampling of the fast relaxing component because of the choice or number of TEs. In this case, only an aggregate relaxation rate is apparent^13^, ^14^ making single compartment fitting appropriate. However, the apparent relaxation rate of this single compartment would depend on the size and specific relaxation rates of the multiple underlying compartments. It would also depend on the flip angle (α) of the measurement because this, together with the compartment‐specific relaxation rates and TR, would dictate the amplitude of the sub‐voxel contributions to the overall measured signal from the voxel.^4^, ^15^, ^16^, ^17^, ^18^ As the exchange rate lowers, multi‐exponential behavior, with distinct compartment‐specific relaxation rates, can be observed.^4^ Each compartment may also have distinct frequency shifts that alter the net signal originating from the voxel.^19^ In white matter (WM), this phenomenon can be modeled by the hollow cylinder fiber model,^20^, ^21^, ^22^, ^23^ which approximates myelinated axons as infinitely long hollow cylinders of myelin, surrounded by and containing non‐myelin water and oriented at a certain angle (θ) with respect to the main magnetic field, B_0_. The difference in isotropic and anisotropic susceptibility of the myelin sheath with respect to the water compartments generates quadratic frequency offsets in the water compartments that depend on θ. With this model, Wharton and Bowtell^21^ derived an approximation predicting a $\sin^{4}\left(\theta \right)$ dependence of $R_{2}^{*}$ on orientation. Although multi‐compartment models are highly appealing for their microstructural specificity, they can have limited validity and/or require a rich array of data for reliable estimation and meaningful precision.^16^, ^24^, ^25^, ^26^

In this work, we used simulation and experiment to investigate the impact that the true multi‐compartment nature of tissue microstructure has on mono‐exponential $R_{2}^{*}$ estimates obtained in vivo in the human brain at 7T. Bloch‐McConnell equations were used to simulate exchanging myelin and non‐myelin water compartments. The impact of myelin water fraction (MWF) and residency time on single compartment $R_{2}^{*}$ estimates, under both ideal and realistic SNR conditions, was quantified. Based on these simulations, we introduced a heuristic linear model of flip angle dependence that partitions the $R_{2}^{*}$ estimates into α‐dependent and α‐independent components. We empirically verify the suitability of this model by applying it to in vivo multi‐parameter mapping (MPM) data. The MPM protocol consists of multi‐echo acquisitions obtained at multiple flip angles and is popular in neuroscientific studies because it can provide a comprehensive set of quantitative MRI parameters with whole brain coverage and high resolution in clinically feasible scan times.^27^, ^28^, ^29^ Within this MPM context, we compared the relative robustness of single compartment $R_{2}^{*}$ estimates, the derived α‐independent component of $R_{2}^{*}$ and a previously established single compartment $R_{2}^{*}$ estimate that pools across multiple flips angles (ESTATICS).^15^ The relative robustness of these estimates to the choice of flip angles, WM fiber orientation, and across measurement sessions was assessed.

## 2 METHODS

### 2.1 Simulating the sensitivity of $R_{2}^{*}$ to multiple compartments

Two compartments,^30^ assumed to be a fast relaxing myelin‐water (MW) compartment ($T_{1,\mathrm{MW}}\;$= 280 ms, $T_{2,\mathrm{MW}}$ = 8 ms)^16^, ^31^, ^32^ and a slower relaxing non‐myelin compartment (i.e., intra‐ and extra‐cellular [IE]; $T_{1,\mathrm{IE}}$ = 1450 ms, $T_{2,\mathrm{IE}}\;$= 36 ms),^21^, ^30^ were simulated using the Bloch‐McConnell equations implemented using the EPG‐X formalism.^33^ A spoiled gradient recalled (SPGR) signal was simulated by including diffusion‐driven spoiling, a net dephasing of 6 ππ per TR, and RF spoiling with an increment of 144°.^34^ Magnetization transfer (MT) effects were not included in the simulations. The net SPGR signal (S_net_) was calculated as:

(1) $$S_{\mathrm{net}}\left(t\right)=\mathrm{abs}\left(S_{\mathrm{MW}}\;e^{-t\;R_{2,\mathrm{MW}}^{*}}+S_{\mathrm{IE}}\;e^{-t\;R_{2,\mathrm{IE}}^{*}}\right).$$

Where *S*_MW_ and *S*_IE_ are the signals of the myelin and non‐myelin water compartments at *t* = 0, respectively, obtained from the EPG‐X simulations, whereas $R_{2,\mathrm{MW}}^{*}$ and $R_{2,\mathrm{IE}}^{*}$ were approximated as $1/T_{2,\mathrm{MW}}$ and $1/T_{2,\mathrm{IE}}$ respectively.

Net SPGR signals were simulated with a TR of 19.50 ms and different flip angles (*α* = [6 9 12 15 19 26 31 36 42]°) and for a range of MWF between 0.02 and 0.20 with a step size of 0.02 representing different tissue conditions (encompassing WM at MWF_WM_ = 0.16 and gray matter [GM] at MWF_GM_ = 0.06).^30^ Different exchange regimes were investigated by varying a directional (MW to IE) residency time between 100 and 500 ms in steps of 100 ms. A single compartment $R_{2}^{*}$ was computed, with log‐linear fitting across TE = (2.56:2.38:14.46) ms of *S*_net_(*t*), for every simulated tissue condition (i.e., each MWF and residency time combination). Simulation parameters are summarized in Supporting Information Table S1. Noisy simulated signals were generated by computing 10 000 instantiations of complex random noise added to the simulated signal with SNR (TE = 0) of 50 to investigate the variance of the $R_{2}^{*}$ estimate.

To investigate the effects of compartment‐specific frequency offsets as described by the hollow cylinder fiber model,^20^, ^21^ we carried out additional simulations taking into account different fiber orientations (θ) with respect to B_0_, ranging from 0° to 90° (with 10° intervals), for each of the 9 flip angles.

### 2.2 Linear model describing $R_{2}^{*}\;$ dependence on α

**FIGURE 1:**
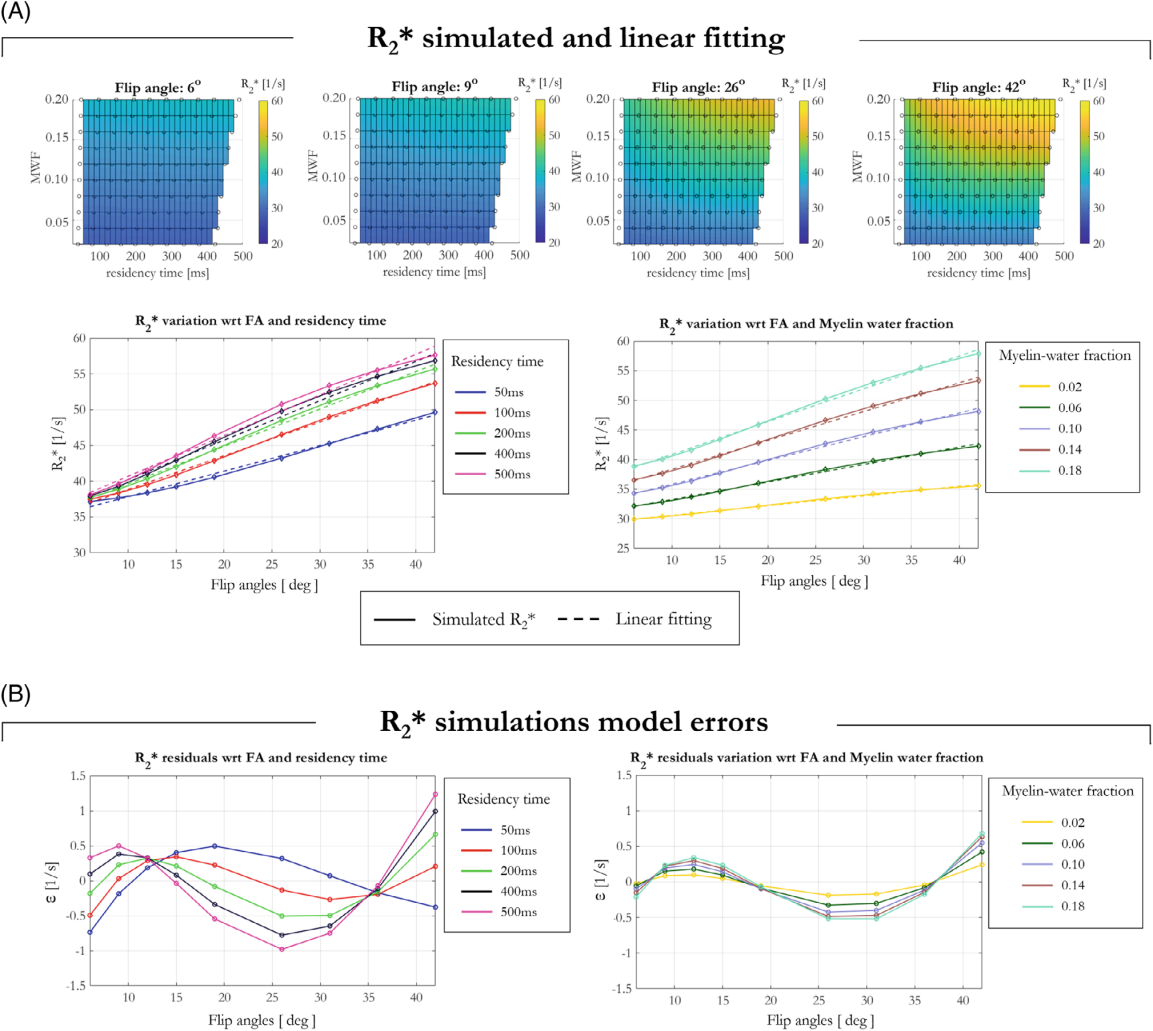
$R_{2}^{*}$ computed via simulations for different residency time, MWF, and flip angles for the case of a fiber oriented parallel to B_0_ (i.e., θ of 0°). (A) Increasing $R_{2}^{*}$ as function of α is observed. (B) A linear model fits the simulated data well as evidenced by model errors <1 s^−1^.

The effective single compartment $R_{2}^{*}$ increases with α for different residency time and MWF. A linear model was applied to the simulated $R_{2}^{*}$ values to partition the α‐independent ($\hat{R_{2}^{*}}\;$) and *α*‐dependent $\left(\frac{dR_{2}^{*}}{d\alpha }\right)$ components, that is:

(2) $$R_{2}^{*}\left(\alpha \right)=\hat{R_{2}^{*}}+\frac{dR_{2}^{*}}{d\alpha }\;\alpha +\varepsilon ,$$

where ε is the model error, which was computed for every simulated tissue property (Figure 1). The model parameters $(\hat{R_{2}^{*}}\;$ and $\frac{dR_{2}^{*}}{d\alpha })$ were estimated by minimizing the model error via least squares estimation. RMSE and RMS percentage error of $R_{2}^{*}$ were calculated for each α across tissues conditions (indexed by *c*, e.g., myelin water fraction and residency time), as:

(3a) $$\text{RMSE}\;R_{2}^{*}=\sqrt{\frac{\sum_{c=1}^{C}\varepsilon_{c}^{2}}{C}}$$

and

(3b) $$\mathrm{RMS}\;\text{percentage error}\;R_{2}^{*}=\sqrt{\frac{\sum_{c=1}^{C}{\left(\frac{\varepsilon_{c}}{R_{2}^{*}{\text{simu}}_{c}}*100\right)}^{2}}{C}}.$$

**FIGURE 2:**
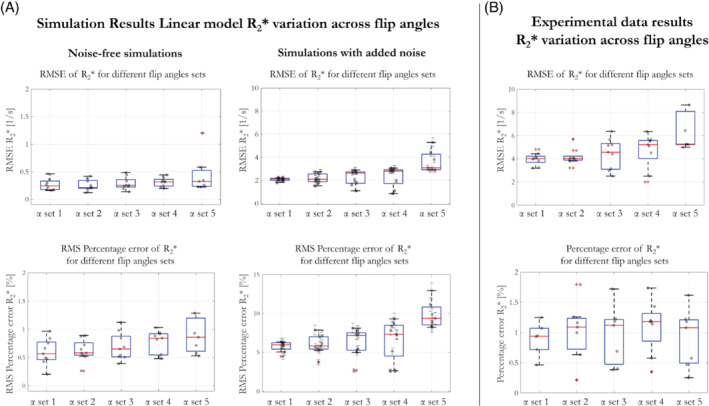
(A) Simulated R_2_* RMSE and RMS percentage error as a function of different α sets with and without noise. Each α set used a different number of flip angles (ranging from 9° to 42°) to estimate the first order model parameters. The points in the bar plot represent the mean error across tissue conditions (MWF and residency time) for each of the 9 flip angles. In the simulations with added noise RMS error was calculated for 10 000 instantiations of noise, therefore, the error bars represent the standard deviation across noise instantiations. (B) RMSE and RMS percentage error from experimental in vivo data at 7T. The same flip angle sets were used in the experiments and simulations.

The robustness of the linear model to the available flip angles was investigated by estimating the RMSE and RMS percentage error of $R_{2}^{*}$ from numerical simulations ($R_{2}^{*}\text{simu})$ and in vivo data (Figure 2). In simulations the RMSE and RMS percentage error were calculated between the $R_{2}^{*}$ estimated on a per flip angle basis via log‐linear fitting of the simulated data across TE, and the per flip angle $R_{2}^{*}$ estimated following application of the proposed linear model of the flip angle dependence. In keeping with the simulations, the in vivo RMS error was calculated from the residuals of the linear model of $R_{2}^{*}$ dependence on flip angle, that is, the difference between the mean measured $R_{2}^{*}$ and the mean $R_{2}^{*}$ estimated with the linear model for a given flip angle.

Different α sets were chosen to encompass a wide range of α (6°‐ 42°), but also to explore the effect of reducing the amount of data available for the fitting of the linear model (decreasing number of α from set 1 to 5). Set 5 is typical of an MPM protocol where the flip angles are chosen to maximize the precision of the subsequently computed R_1_ estimates.^29^, ^35^ Using the fitted $\hat{R_{2}^{*}}$ and $\frac{dR_{2}^{*}}{d\alpha }$ values, an approximated $R_{2}^{*}$ was estimated for each of the 9 flip angles and model residuals were calculated in vivo for comparison with the simulation results.

- α set 1 = [6, 9, 12, 15, 19, 26, 31, 36, 42]°
- α set 2 = [6, 9, 15, 26, 31, 42]°
- α set 3 = [6, 9, 2,6 42]°
- α set 4 = [6, 26, 42]°
- α set 5 = [6, 26]°

**FIGURE 3:**
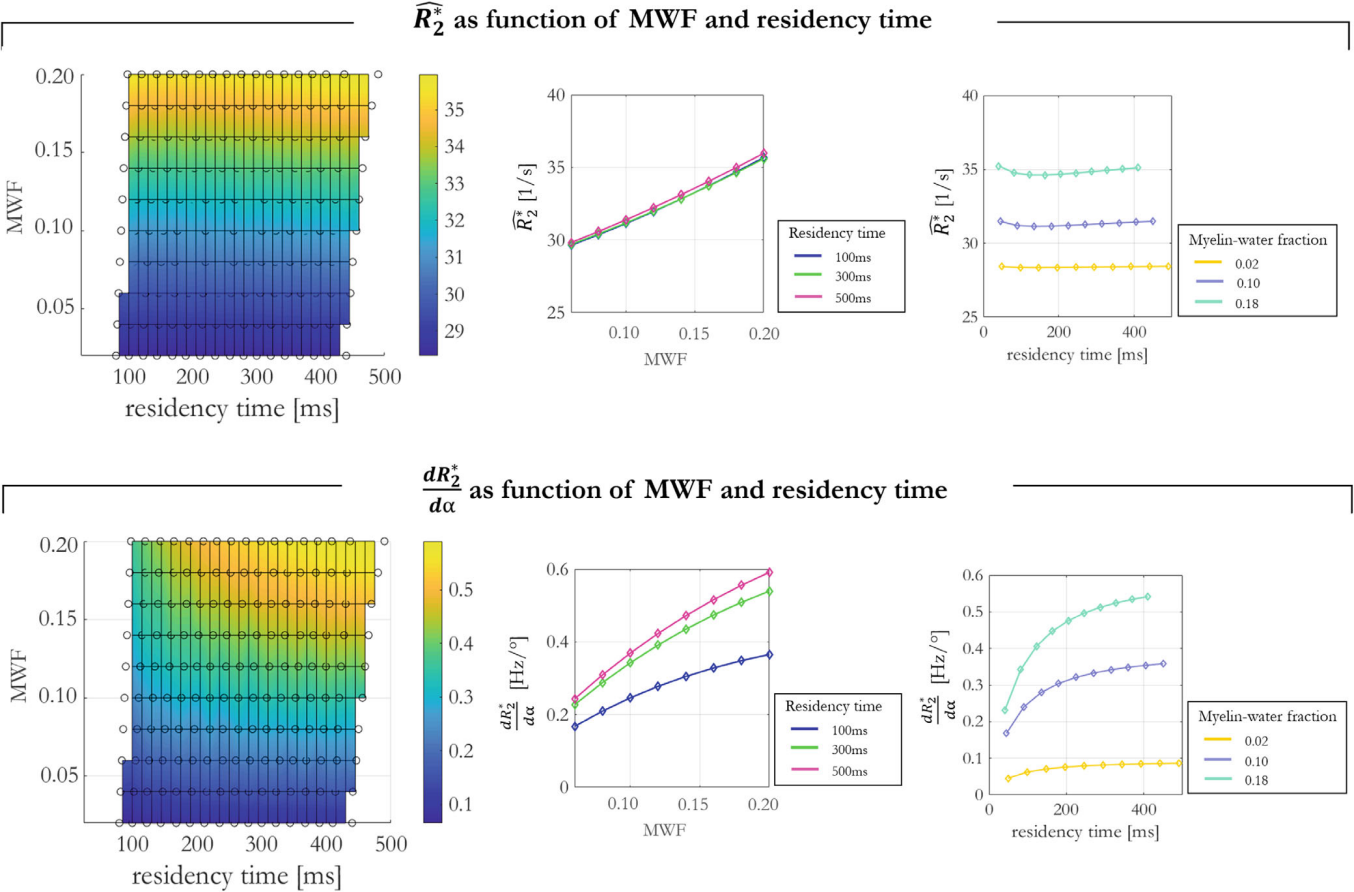
$\hat{R_{2}^{*}}\;$ and $\frac{dR_{2}^{*}}{d\alpha }\;$ dependence on MWF and residency time. The α‐independent component, $\hat{R_{2}^{*}},$ had a sensitivity of 12.6% to MWF, but only 0.74% sensitivity to residency time. The α‐dependent component $\frac{dR_{2}^{*}}{d\alpha }$ had a sensitivity of 13% to residency time and 55% sensitivity to MWF.

Finally, the sensitivity of $\hat{R_{2}^{*}}\;$ and $\frac{dR_{2}^{*}}{d\alpha }$ to different tissue conditions was investigated and summarized as the maximum variation over the mean of $\hat{R_{2}^{*}}\;$ and $\frac{dR_{2}^{*}}{d\alpha }$, respectively (Figure 3).

### 2.3 Acquisitions

#### 2.3.1 MPM protocol

Data were acquired on a Siemens 7T Terra (Siemens Healthcare, Erlangen, Germany) using a head coil with 8 transmit and 32 receive channels (Nova Medical, Wilmington, Massachusetts, USA). The MPM protocol^29^ closely matched the settings used in the simulations. It comprised 3 multi‐echo 3D SPGR scans acquired with T1 (*α*_T1w_ = 26°), proton density (PD) (*α*_PDw_ = 6°) or magnetisation transfer (MT) (*α*_MTw_ = 6°) weighting. Six echoes were acquired with TE ranging from 2.56 to 14.46 ms in steps of 2.38 ms using a TR of 19.5 ms. Only the first 4 echoes were acquired for the MT‐weighted scan to allow time for the pre‐pulse (Gaussian‐shaped RF pre‐pulse with 4 ms duration, 180° nominal flip angle, 2 kHz frequency offset from water resonance). Imaging parameters included FOV of 192 × 192 × 160 mm^3^ with 1 mm isotropic resolution, spoiler gradient moment of 6π per TR, RF spoiling increment of 144°.^36^ Partial Fourier (6/8) in both phase‐encoding directions and elliptical sampling were used to achieve a single scan duration of 5 min. This core MPM protocol was extended for the acquisition of additional multi‐echo 3D SPGR scans with flip angles of 9°, 12°, 15°, 19°, 31°, 36°, and 42°, for a total of 10 acquired 3D datasets (9 α and 1 MTw).

An in‐house sequence exploiting the Bloch‐Siegert shift was used to map the effective transmitting field (${B_{1}^{+}}_{\mathrm{eff}}$).^37^ Relevant parameters included: single echo, TE/TR = 6.77/40 ms, 14° flip angle, FOV of 256 × 256 × 192 mm^3^ with 4 mm isotropic resolution, using a Fermi pulse with an off‐resonance frequency of ±2 kHz and 4‐ms duration to impart the Bloch‐Siegert phase that encodes ${B_{1}^{+}}_{\mathrm{eff}}$.

#### 2.3.2 In vivo $R_{2}^{*}$ and $\hat{R_{2}^{*}}$ estimation for correction of the flip angle bias

The hMRI toolbox^28^ was used to process each variable flip angle (VFA) MPM dataset. $R_{2}^{*}$ maps for each nominal α (for a total of 9 maps) were estimated with a voxel‐wise log‐linear fit across TE.

Three additional $R_{2}^{*}$ maps were reconstructed with the ESTATICS^15^ approach by combining flip angle pairs: [6°, 26°], [9°, 42°] and [9°, 26°]. ESTATICS pools multi‐echo data from each α acquisition and performs a single log‐linear fit assuming a common decay, resulting in $R_{2}^{*}$ maps with enhanced SNR (Supporting Information Figure S1). $\hat{R_{2}^{*}}$ maps were also generated for each of the α pairs used to estimate ESTATICS $R_{2}^{*}$ maps.^15^

$\hat{R_{2}^{*}}$ and $\frac{dR_{2}^{*}}{d\alpha }$ maps were generated by fitting the linear model in Equation (2) voxel‐wise, incorporating transmit field inhomogeneity (${B_{1}^{+}}_{\mathrm{eff}})$, such that:

(4) $$R_{2}^{*}(r)=\hat{R_{2}^{*}}(r)+\frac{dR_{2}^{*}}{d\alpha }\;(r)\alpha \;{B_{1}^{+}}_{\mathrm{eff}}(r)+\varepsilon (r).$$

Where *r* indicates the spatial location of each voxel.

#### 2.3.3 DWI

To investigate the $R_{2}^{*}$, $\hat{R_{2}^{*}}$, and $\frac{dR_{2}^{*}}{d\alpha }$ dependence on different WM fiber orientations, we used DWI to determine the first eigenvector of the diffusion tensor (representing local fiber orientation). DWI were acquired at 3T (Siemens Prisma, Siemens Healthcare, Erlangen, Germany) using an EPI acquisition with multi‐band factor of 2 and 151 diffusion encoded directions with 4 interleaved *b*‐values of 0, 500, 1000, and 2300 s/mm^2^. Imaging parameters were as follows: FOV of 220 × 220 × 144 mm^3^ with 2 mm isotropic resolution, TE/TR = 60/3320 ms and flip angle = 88°. Additional data were acquired with no diffusion encoding but reversed polarity of the phase‐encoding gradients to facilitate correction of susceptibility‐induced distortions. The ACID toolbox (http://diffusiontools.com/) was used to process the diffusion data. Pre‐processing of the diffusion data included 3 steps:

1.
Affine registration of the diffusion dataset to correct for the misalignment caused by motion and eddy currents.^38^
2.
Multi‐shell position‐orientation adaptive smoothing, which reduces the noise of the acquired data without blurring tissue boundaries.^39^
3.
Hyperelastic susceptibility artifact correction, which exploits the reversed gradient‐based acquisition scheme to remove distortion artifacts.^40^

The pre‐processed data were fitted with a non‐linear least squares diffusion kurtosis model to obtain a fractional anisotropy map, and an angle map describing the WM fiber orientation with respect to B_0._ ^41^, ^42^

#### 2.3.4 Imaging sessions

Three healthy volunteers (female, 42 year‐old [participant 1], male, 40 year‐old [participant 2] and female, 34 year‐old [participant 3]) were scanned at 7T (Siemens Terra) across a total of 6 imaging sessions, which are summarized in Supporting Information Table S2. Approval was obtained from the local research ethics committee and written informed consent was obtained from each participant before scanning.

To evaluate the effects of different *α* on $R_{2}^{*}$ in vivo, the extended VFA MPM data were acquired.

Long‐term and short‐term reproducibility was analyzed by acquiring data at 7T in 3 scan sessions on participant 1, after 1 year (session 1: 11/02/2020; session 2: 10/02/2021) and 1 week (session 3: 17/02/2021), respectively.

The DWI data were acquired in a separate session on participant 1 to assess $R_{2}^{*}$, $\hat{R_{2}^{*}}$, and $\frac{dR_{2}^{*}}{d\alpha }$ dependence on WM fiber orientation.

### 2.4 Data analysis

All images were analyzed using SPM12 (http://www.fil.ion.ucl.ac.uk/spm/software/spm12/, Wellcome Centre for Human Neuroimaging, London, UK).

The T_1_‐weighted images, with α = 26°, for each participant and each session were co‐registered to session 1 and segmented. Participant and session specific WM and GM masks were defined by those voxels with a probability of belonging to the respective tissue class >0.9. A single participant‐specific WM or GM mask was obtained by combining the WM or GM masks across sessions via logical conjunction. To ensure equivalent processing, each $R_{2}^{*}$, $\hat{R_{2}^{*}}$, and $\frac{dR_{2}^{*}}{d\alpha }$ map was co‐registered to the T_1_‐weighted image acquired with α = 26° in session 1 for each participant.

Reproducibility of $R_{2}^{*}$ quantification was evaluated for each participant for: single α $R_{2}^{*}$, ESTATICS and $\hat{R_{2}^{*}}$ approaches. Single α: 6°, 26°, or 42° and α‐pairs: [6°, 26°], [9°, 42°], or [9°, 26°] were used to compute the $R_{2}^{*}$ estimates for this analysis. $R_{2}^{*}$ reproducibility among different α (or α pairs for ESTATICS and $\hat{R_{2}^{*}}$ approaches) was quantified by the coefficient of variation (COV). COV_FA_ was defined as the $R_{2}^{*}$ standard deviation across α relative to the mean, in percent. This was computed separately for GM and WM and summarized per tissue by taking the median across voxels (Equation [5], where V = voxels).

(5) $${\mathrm{COV}}_{\mathrm{FA}}={\text{median}}_{V}\left(\;\frac{\mathrm{std}(\mathrm{FA}\;\text{datasets})}{\text{mean}(\mathrm{FA}\;\text{datasets})}\;100\right).$$

$R_{2}^{*}$ repeatability across 2 sessions was assessed for participant 1 (session 2 and 3). Bias was defined as the mean across voxels (V) of the median across α pairs, of the voxel‐wise difference between sessions (Equation [6a]). 95% confidential intervals (CI) were defined as 1.96 times the standard deviation across voxels of the median across α pairs of the per‐voxel difference between sessions (Equation [6b]):

(6a) $$\begin{array}{cc} \text{bias session} & ={\text{mean}}_{V}\;({\text{median}}_{\mathrm{FA}\text{pairs}}\\ & \;(\text{difference between sessions})) \end{array}$$

and

(6b) $$\begin{array}{cc} \mathrm{CI}\;\text{session} & =1.96\;{\mathrm{std}}_{V}\;({\text{median}}_{\mathrm{FA}\;\text{pairs}}\\ & \;\left(\text{difference between sessions})\right). \end{array}$$

Bland Altman plots of the voxel‐wise differences across sessions against the mean are shown in Figure 6.

Finally, the per‐α $R_{2}^{*}$, $R_{2}^{*}$ from ESTATICS, $\hat{R_{2}^{*}}$ and $\frac{dR_{2}^{*}}{d\alpha }$ dependence on fiber orientation with respect to B_0_ was analyzed using data from participant 1 from imaging sessions 2 and 3 (acquired at 7T) and are shown in Figure 7.

In the space of the diffusion data, the angle between the fibers and B_0_ is defined as:

(7) $$\theta \left(r\right)=\text{arcos}\left(\frac{\left|v_{z}\left(r\right)\right|}{\left|\vec{v}\left(r\right)\right|}\right).$$

Where *r* represents voxel location, $\vec{v}$ is the fiber orientation in the diffusion data space and $v_{z}$ is the orientation component aligned with B_0._ ^21^

The $R_{2}^{*}$ maps for each scanning session were first resliced into the diffusion space (accounting for differences in FOV positioning and angulation). Subsequently, an affine transformation between the diffusion data and the maps was determined via co‐registration to account for any head rotation between the different scanning sessions. The resulting transformation was applied to the primary fiber orientation directions ($v_{x}$, $v_{y}$, $\;\text{and}\;v_{z}$) in diffusion space and a new angle map, which represents the angle between the fiber orientation and B_0_ at each head position (different scanning sessions), was computed with Equation (7).

$R_{2}^{*}\left(r\right)$, $\hat{R_{2}^{*}}\left(r\right)$, $\frac{dR_{2}^{*}}{d\alpha }\left(r\right)$, and θ(r) measurements were extracted in WM voxels with a WM probability >0.9 and a fractional anisotropy >0.6. The fiber angles θ(r) were segregated into bins containing 200 voxels to have a sufficient number for calculation of reliable summary statistics. For each bin, mean $R_{2}^{*}\left(r\right)$, $\hat{R_{2}^{*}}\left(r\right)$, and $\frac{dR_{2}^{*}}{d\alpha }\left(r\right)$ were calculated and plotted against θ(r). The function $R_{2}^{*}\left(\theta \right)=R_{2,\mathrm{Iso}}^{*}+R_{2,\text{Aniso}}^{*}\;\sin^{4}\left(\theta \right)$, predicted by the hollow cylinder fiber model,^20^, ^43^ was fit to the data to extract the isotropic component of $R_{2}^{*}$ ($R_{2,\mathrm{Iso}}^{*}$) and the proportional θ‐dependence via $R_{2,\text{Aniso}}^{*}/R_{2,\mathrm{Iso}}^{*}$. Per single‐α $R_{2}^{*}$ was computed for α = 26° whereas ESTATICS, $\hat{R_{2}^{*}}$, and $\frac{dR_{2}^{*}}{d\alpha }$ were computed for the α pair = [6, 26]°.

The reproducibility of the $R_{2}^{*}$, ESTATICS, and $\hat{R_{2}^{*}}$ isotropic components and θ‐dependence $\left(R_{2,\text{Aniso}}^{*}/R_{2,\mathrm{Iso}}^{*}\right)$ across α and α‐pairs (for ESTATICS and $\hat{R_{2}^{*}}$ approaches) was computed as COV_*α*_ defined as:

(8) $${\mathrm{COV}}_{\alpha }=\frac{\mathrm{std}\left(\alpha \;\text{datasets}\right)}{\text{mean}\left(\alpha \;\text{datasets}\right)}\;100.$$

The single α and α‐pairs considered in the analysis were: 6°, 26°, 42° (for $R_{2}^{*}$ estimates), and [6, 26]°, [9, 42]°, and [9, 26]° (pairs suitable for R_1_ mapping) for the estimates obtained with ESTATICS and $\hat{R_{2}^{*}}$.

Reproducibility of $R_{2,\mathrm{Iso}}^{*}$ and $R_{2,\text{Aniso}}^{*}/R_{2,\mathrm{Iso}}^{*}$ across 2 sessions (session 2 and 3) was measured as bias ± CI. Bias and 95% CI were respectively quantified as the mean and standard deviation (scaled by 1.96) across α or α‐pairs of the difference of estimates between sessions.

## 3 RESULTS

### 3.1 Simulation results

Simulating $R_{2}^{*}$ for 9 different α and for tissues properties spanning MWF = 0.02:0.02:0.20 and residency time = 100:100:500 ms revealed an increase in $R_{2}^{*}$ as a function of α for every tissue condition, as shown in Figure 1A for 4 representative flip angles. The linear model shown in Equation (2) was used to fit the data. Good agreement was observed between the simulated data and the linear model fit, as evidenced by model errors <1 s^−1^ (Figure 1B).

Simulations investigating the effects of the compartment‐specific frequency offsets showed that the linear model (Equation [2]) approximated the data well for every simulated fiber orientation. Supporting Information Figure S2 shows the $R_{2}^{*}$ computed for each θ and α via log‐linear fitting across TE (solid lines with diamonds), $\hat{R_{2}^{*}}$ computed via linear fitting pooling all flip angles for a given θ (turquoise solid line with circles) and $R_{2}^{*}$ estimated following application of the proposed linear model of the flip angle dependence (dashed lines). Good agreement was observed between the simulated data and the linear model fit for every flip angle (solid and dashed lines).

Simulations probing the dependence of the flip angle dependent $\left(\frac{dR_{2}^{*}}{d\alpha }\right)$ and flip angle independent ($\hat{R_{2}^{*}}$) components of $R_{2}^{*}$ on MWF and residency times were repeated for θ = 90°. These results are shown in Supporting Information Figure S3, and are in keeping with those of Figure 1, which depicts the equivalent results for θ = 0°. $R_{2}^{*}$ increased with MWF and residency time. Furthermore, the proposed linear model continues to fit the data well, evidenced by residuals <2 Hz. $R_{2}^{*}$ also increased with θ, an effect that was accentuated at higher flip angles (Supporting Information Figure S2), whereas $\hat{R_{2}^{*}}$ had a greatly reduced dependence on θ.

RMSE and RMS percentage error of $R_{2}^{*}$ were computed for each α and collapsed across tissue conditions (Figure 2). This was done for 5 different α sets, containing variable numbers of flip angles, and used to estimate the linear model parameters. In the noise‐free case, RMSE <0.6 s^−1^ and RMS percentage error <1.5% were observed for each α set. The variability of the error, across tissue conditions, increased as the number of α used to fit the linear model decreased. The same trend was observed when noise was added to the simulations. In this case, RMSE increased and ranged from 2.1 to 3.2 s^−1^ going from α set 1 (9 flip angles) to α set 5 (only 2 flip angles), respectively, whereas RMS percentage error increased to 6.2% and 9.1%, respectively. In agreement with simulation, the error in the experimental case for participant 1 (Figure 2B) increased as the number of flip angles included in the computation decreased. Consistent results were obtained for participant 2.

The dependence of the linear model coefficients $(\hat{R_{2}^{*}}\;$ and $\frac{dR_{2}^{*}}{d\alpha }\;)$ on different tissue conditions is shown in Figure 3. The α‐independent component, $\hat{R_{2}^{*}},$ showed a high sensitivity of 12.6% to MWF as it ranged from 0.02 to 0.20. However, it was effectively independent of the residency time (0.74% maximal sensitivity). $\frac{dR_{2}^{*}}{d\alpha }$ depended on both MWF and residency time. It had a maximal sensitivity of 13% to residency time, and a larger maximal sensitivity of 55% to MWF. The latter dependence was approximately quadratic.

### 3.2 In vivo results

**FIGURE 4:**
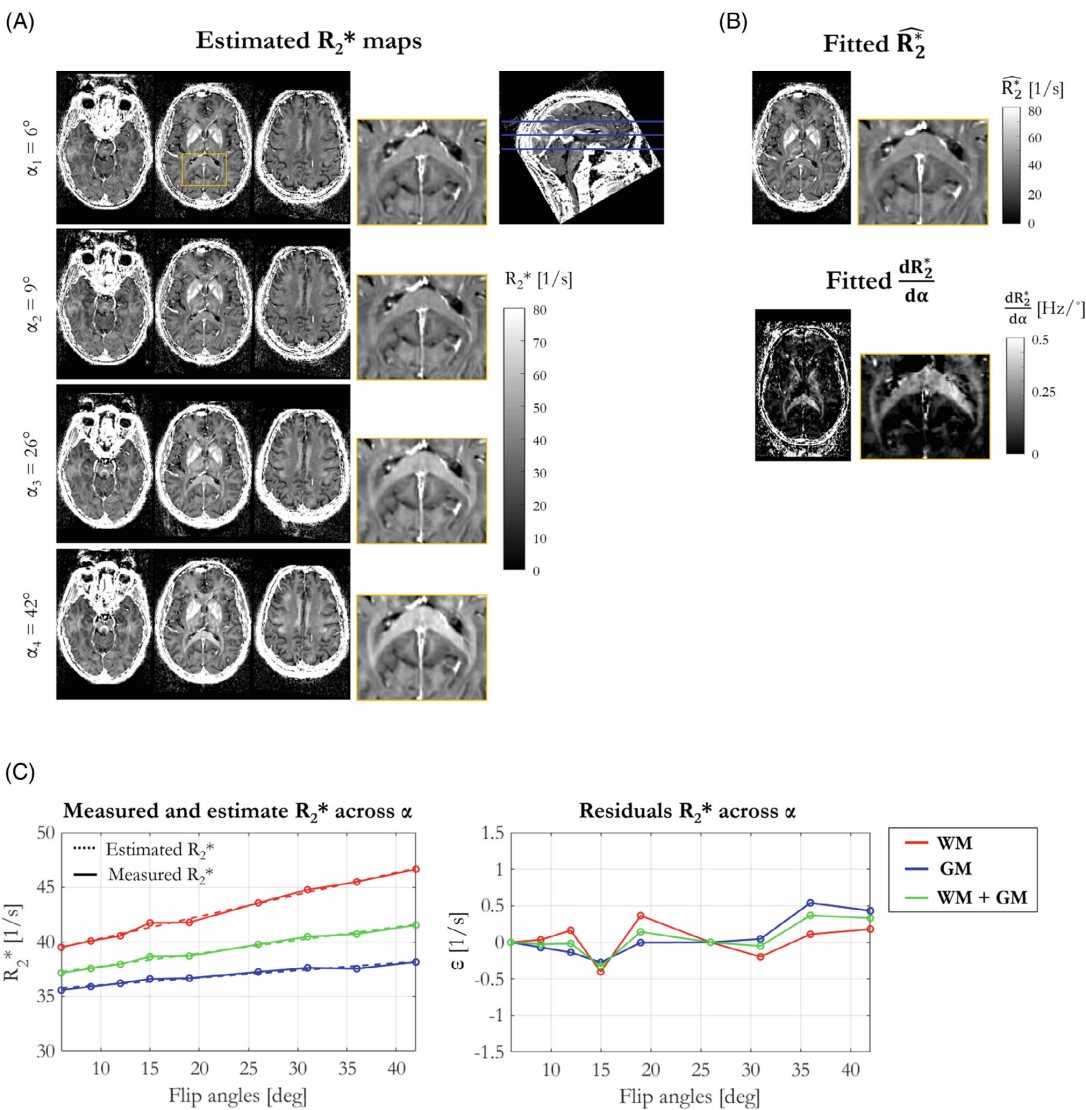
$R_{2}^{*}$ maps were computed for 9 nominal α, 4 representative maps are shown in (A). These experimental data show an increase in $R_{2}^{*}$ with increasing α. (B) α‐independent $\hat{R_{2}^{*}}$ and α‐dependent $\frac{dR_{2}^{*}}{d\alpha }$ components were obtained by fitting the linear model (Equation [4]) to these data. (C) The linear model was used to recompute $R_{2}^{*}$ for each α. The measured and estimated $R_{2}^{*}$ and the model residuals, *ε*, are shown as a function of α.

$R_{2}^{*}$ maps were computed for 9 nominal α. Four representative $R_{2}^{*}$ maps, obtained with α = [6, 9, 26, 42]°, are shown in Figure 4A. An increase in $R_{2}^{*}$ is visually apparent with increasing flip angle, most notably in the corpus callosum (zoomed view). $\hat{R_{2}^{*}}\;$ and $\frac{dR_{2}^{*}}{\mathrm{d\alpha}}$ components obtained by fitting the linear model in Equation (4) are shown in Figure 4B. Consistent results were obtained for all the participants.

Figure 4C shows the mean measured $R_{2}^{*}$ in WM (red solid line), GM (blue solid line), and WM and GM combined (green solid line) obtained across the 9 nominal α. The dashed lines (with colors indicating tissue type as before) show the corresponding mean $R_{2}^{*}$ obtained by fitting the linear model (Equation [4]) in WM, GM, and WM + GM. The mean model residuals were <1 s^−1^ across all α, showing good agreement with simulation results.

**FIGURE 5:**
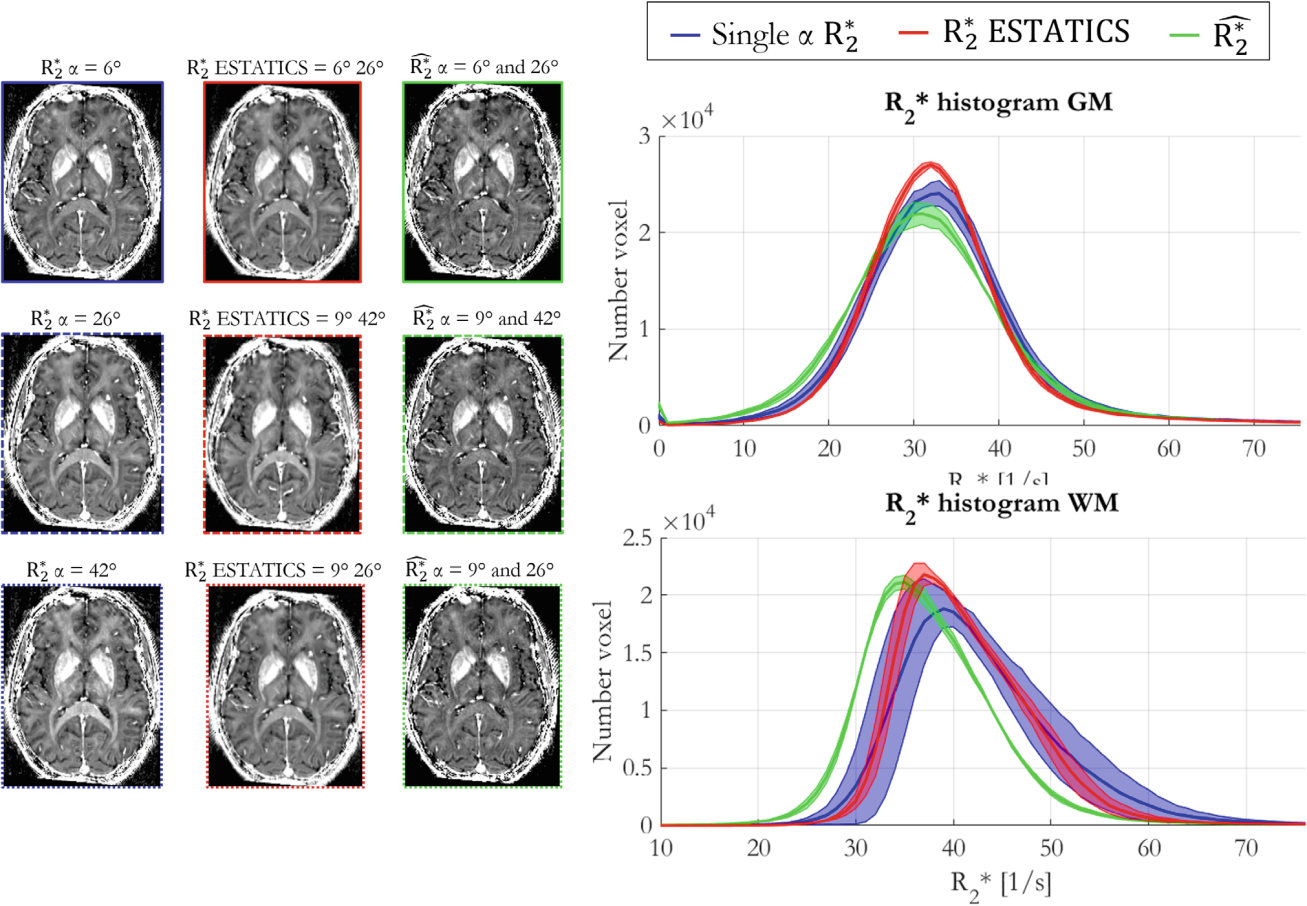
$R_{2}^{*}$ maps obtained with nominal α of 6°, 26°, and 42° (blue) or for α pairs of [6°, 26°], [9°, 42°], and [9°, 26°] using ESTATICS (red) or the α‐independent component of the heuristic linear model (green) are shown on the left. Histograms of the $R_{2}^{*}$ estimates for each α or α‐pair are shown on the right. The solid lines represent the mean across α or α‐pairs, and the shaded area represents the standard deviation.

Figure 5 shows various $R_{2}^{*}$ maps computed using the multi‐echo data obtained with α = 6°, 26° and 42°. These were either fit individually (blue), and subsequently used to derive the α‐independent component ($\hat{R_{2}^{*}}\;$) of the linear model (green) using two α, or the data from two α were combined at the point of fitting $R_{2}^{*}$ using the ESTATICS approach (red). Histograms of values were consistent for $\hat{\;R_{2}^{*}}$, but variable for $R_{2}^{*}$, particularly in WM, when estimated on a per‐α basis instead of using ESTATICS (Figure 5A). The reproducibility analysis, for each of the 3 participants, revealed $\hat{R_{2}^{*}}$ to be most robust to α variation in both WM and GM (Table 1, median COV of 0.98% and 1.42%, respectively), whereas the per‐α $R_{2}^{*}$ estimates were least robust regardless of tissue type (Table 1, median COV of 9.61% and 11.98%, respectively). The robustness of the ESTATICS approach was intermediate (Table 1, median COV of 5.98% and 6.14%, respectively).

**TABLE 1:** Coefficients of variation (COVs) calculated across different α or α‐pairs within a single imaging session for the 3 participants

			$R_{2}^{*}$	$R_{2}^{*}$ ESTATICS	$\hat{R_{2}^{*}}$
Participant 1	Inter α set COV [%]	GM	11.98	2.75	1.33
		WM	9.93	5.98	0.98
Participant 2	Inter α set COV [%]	GM	11.72	6.63	1.42
		WM	9.61	7.24	1.12
Participant 3	Inter α set COV [%]	GM	12.72	6.14	1.87
		WM	8.09	5.69	0.98

**FIGURE 6:**
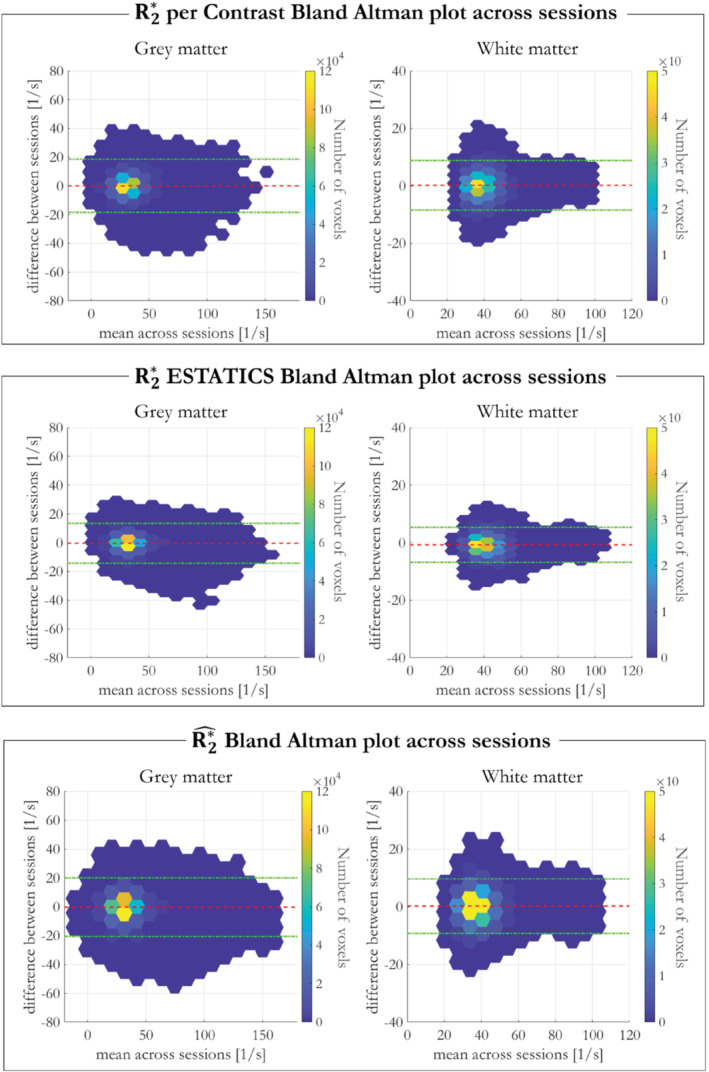
Bland–Altman plots showing the voxel‐wise difference in $R_{2}^{*}$ estimates across sessions as a function of their mean in GM (left) and WM (right). The color scale indicates the voxel density for a certain difference vs mean point. The red lines show the biases and the green lines show the confidence intervals.

Bland–Altman analyses of inter‐session repeatability (participant 1, sessions 2 and 3) are shown in Figure 6, with biases ± CI summarized in Supporting Information Table S3. $\hat{R_{2}^{*}}$ showed the smallest bias in WM, $R_{2}^{*}$ ESTATICS had the smallest bias in GM, whereas per‐α $R_{2}^{*}$ showed the highest biases in both GM and WM. However, the CI was largest for $\hat{R_{2}^{*}}$ indicating poorest cross‐session repeatability.

**FIGURE 7:**
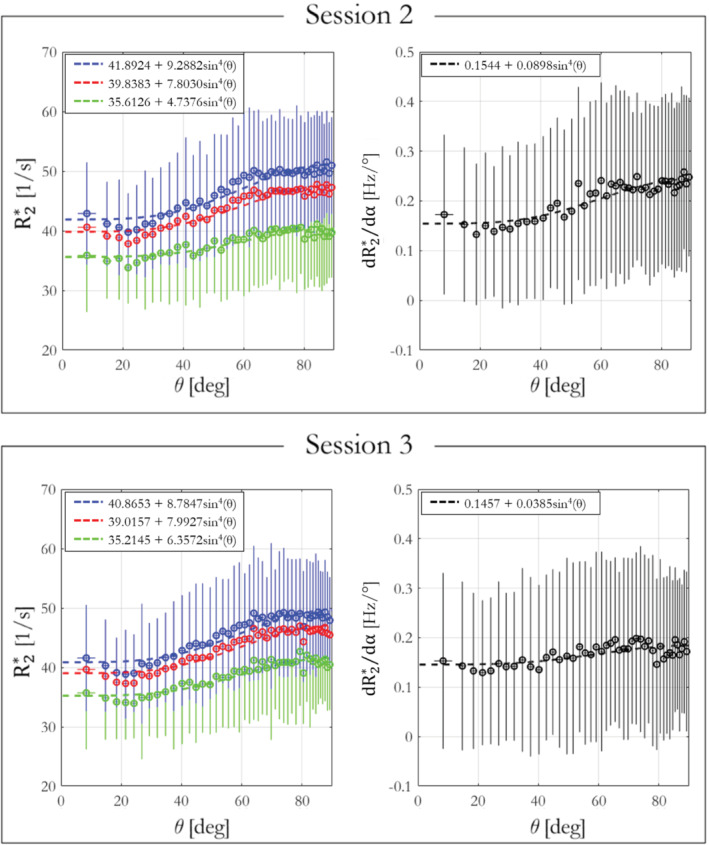
$R_{2}^{*}$ (blue), $\;R_{2}^{*}$ ESTATICS (red) and $\hat{R_{2}^{*}}$ (green) and ${\mathrm{dR}}_{2}^{*}/d\alpha$ (black) dependence on WM fiber orientation with respect to the main field, B_0_, in sessions 2 and 3.

The $R_{2}^{*}$, $R_{2}^{*}$ ESTATICS, $\hat{R_{2}^{*}}$, and $\frac{dR_{2}^{*}}{d\alpha }$ dependence on WM fiber orientation with respect to B_0,_ (θ), is shown in Figure 7 for data acquired in sessions 2 and 3 (orientation data from session 4). The result of fitting the $\sin^{4}\left(\theta \right)$ dependence predicted by the hollow cylinder fiber model is inset. $\hat{R_{2}^{*}}$ (green) had lower θ‐dependence than $R_{2}^{*}$ estimated with a single α (α = 26°, blue) or the ESTATICS approach (red), with the ratio $R_{2,\text{Aniso}}^{*}/R_{2,\mathrm{Iso}}^{*}$ for (session 2, session 3) being (0.133, 0.181), (0.223, 0.215), and (0.198, 0.205), respectively. $\frac{dR_{2}^{*}}{d\alpha }$ showed the highest orientation dependence with $R_{2,\text{Aniso}}^{*}/R_{2,\mathrm{Iso}}^{*}$ of 0.5815 and 0.264 in sessions 2 and 3, respectively. This observation agrees with simulations where the $R_{2}^{*}$ θ‐dependence is greatly reduced in $\hat{R_{2}^{*}}$ and therefore must propagate into the $\frac{dR_{2}^{*}}{d\alpha }$ component. $R_{2}^{*}$ depends not only on orientation, but also on the spatially varying microstructural composition of the tissue. Despite this, the dependence on fiber orientation predicted by the hollow cylinder fiber model is apparent in the data—as is the fact that this dependence increases with flip angle (Supporting Information Figure S4). Good agreement, in terms of pattern and effect size, was found between simulations (Supporting Information Figure S2) and in vivo results of $R_{2}^{*}$ and $\hat{R_{2}^{*}}$ dependence on θ (Supporting Information Figure S4).

**FIGURE 8:**
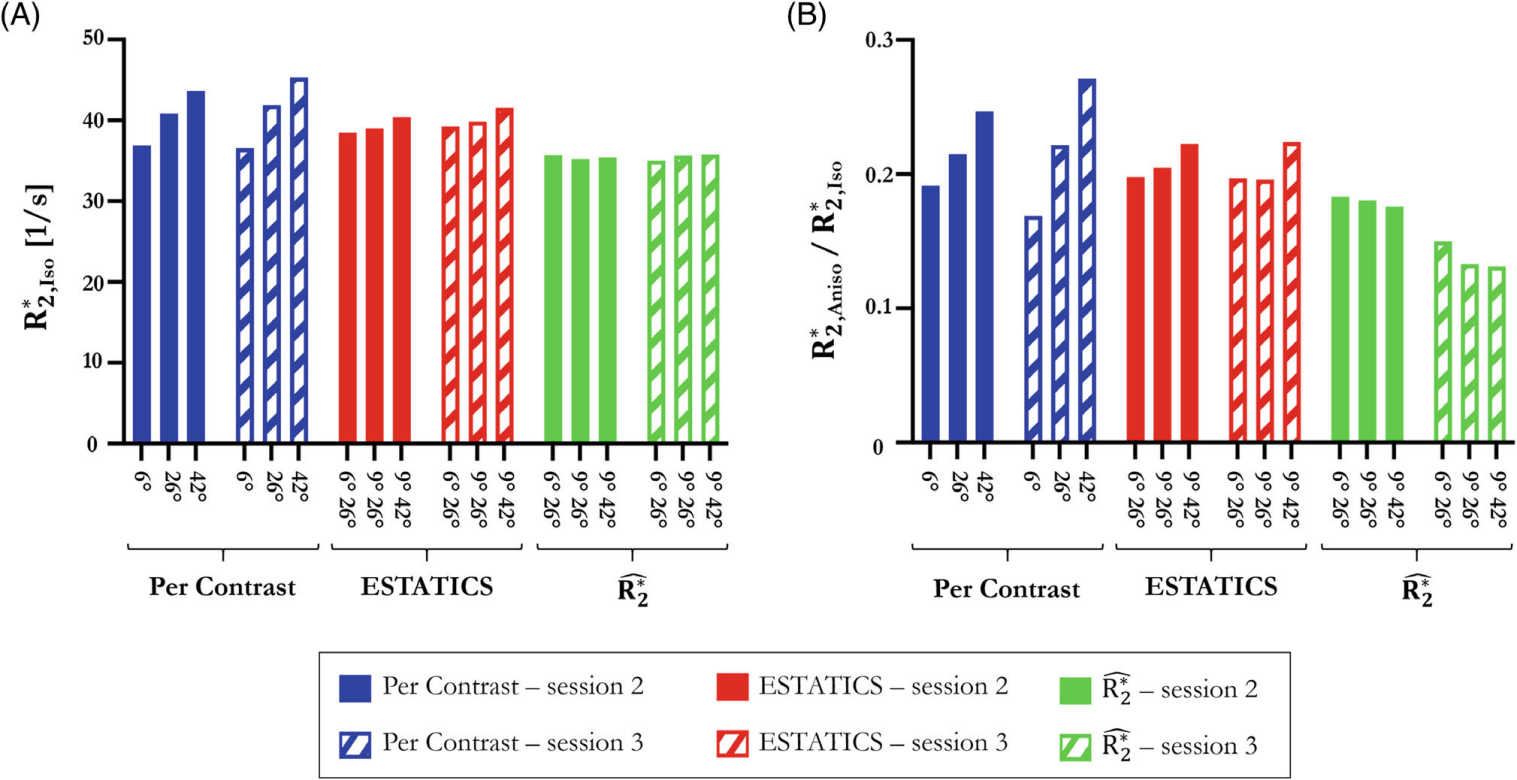
The dependence of $R_{2}^{*}$ on fiber orientation was decomposed into orientation independent ($R_{2}^{*}$_,iso_) and dependent ($R_{2,\text{Aniso}}^{*}$) components. Reproducibility across sessions and as a function (A) of α for $R_{2}^{*}$_,iso_ and (B) proportional fiber orientation dependence quantified by $R_{2,\text{Aniso}}^{*}/R_{2,\mathrm{Iso}}^{*}$ obtained with single‐α fits, or using α pairs in the ESTATICS and proposed linear modeling approache.

Figure 8 summarizes theθθ‐dependence of the $R_{2}^{*}$ estimates across 2 sessions. $R_{2,\mathrm{Iso}}^{*}$ was most robust, across both α‐pairs and sessions, when derived from $\hat{R_{2}^{*}}$ (Figure 8A ) (Table 2 , COV _*α*_ = 0.74%, Inter‐session bias ± CI = −0.006 ± 0.980). $\hat{R_{2}^{*}}$ was also least θ‐dependent (lowest $R_{2,\text{Aniso}}^{*}/R_{2,\mathrm{Iso}}^{*}$) (Figure 8B ) and the most consistent as α varied (COV _*α*_ = 3.88% versus 5.66% for ESTATICS and 14.70% for $R_{2}^{*}$ per‐α). However, the anisotropic component derived from $\hat{R_{2}^{*}}$ had the lowest cross‐session reproducibility (inter‐session bias ± CI = 0.041 ± 0.010).

**TABLE 2:** COV across different α sets and biases between different imaging sessions for the fiber orientation independent ($R_{2}^{*}$_,iso_) and dependent ($R_{2,\text{Aniso}}^{*}$/$R_{2}^{*}$_,iso_) components of $R_{2}^{*}$

	Per contrast		ESTATICS		$\hat{R_{2}^{*}}$	
	$R_{2,\mathrm{Iso}}^{*}$	$\frac{R_{2,\text{Aniso}}^{*}}{R_{2,\mathrm{Iso}}^{*}}$	$R_{2,\mathrm{Iso}}^{*}$	$\frac{R_{2,\text{Aniso}}^{*}}{R_{2,\mathrm{Iso}}^{*}}$	$R_{2,\mathrm{Iso}}^{*}$	$\frac{{\hat{R_{2}^{*}}}_{,\mathrm{Iso}}}{{\hat{R_{2}^{*}}}_{,\text{Aniso}}}$
Inter α set COV [%]	7.78	14.72	2.25	5.66	0.74	3.88
Inter session bias ± CI	−0.802 ± 1.16 [1 s^−1^]	−0.002 ± 0.04 [n.a.]	−0.913 ± 0.34 [1 s^−1^]	0.003 ± 0.01 [n.a.]	−0.006 ± 0.98 [1 s^−1^]	0.041 ± 0.01 [n.a.]

## 4 DISCUSSION

In this work, we investigated both numerically and empirically, how the true multi‐compartment nature of human brain tissue manifests in single compartment $R_{2}^{*}$ estimates typical of neuroscientific studies. We focused on the exchanging myelin and intra‐extracellular water compartments, which have differential contribution to the measured signal as α varies causing $R_{2}^{*}$ to also depend on α. Our simulations showed that the α‐dependence increased with MWF and residency time. We introduced an efficient linear model to correct for this $R_{2}^{*}$ α‐dependence and demonstrated its robustness in simulations and in vivo experiments. We assessed, in simulations and empirically, the orientation dependence of the α‐independent component of the linear model ($\hat{R_{2}^{*}}$) as well as its reproducibility across sessions in comparison to the per‐α or ESTATICS counterparts. $\hat{R_{2}^{*}}$ has appealing robustness to flip angle and orientation, but comes with a modest increase in noise sensitivity.

The two compartment simulations, across a wide range of MWF and residency times, using the Bloch‐McConnell equations, replicated the α‐dependence of $R_{2}^{*}$ estimates observed in vivo. With a linear model of the α‐dependence, we observed excellent agreement between the empirical observations (Figure 4) and simulations despite the highly simplifying assumption of just two exchanging water pools (Figure 1). The fitted offsets predicted by simulation (Figure 1B) were in line with those obtained in vivo in GM and WM (Figure 4C).

The linear model partitions the effective transverse relaxation into α‐independent ($\hat{R_{2}^{*}}$) and α‐dependent $\left(\frac{dR_{2}^{*}}{d\alpha }\right)$ components. In agreement with simulations, $\hat{R_{2}^{*}}\;$ robustly removed the α‐dependence in vivo, and as a result showed higher reproducibility across α sets than $R_{2}^{*}$ estimated on a per‐α basis or with ESTATICS (Figure 5). Empirically, the dependence of $\hat{R_{2}^{*}}$ on the WM fiber orientation with respect to B_0_ was also reduced in comparison to $R_{2}^{*}$ estimated either via a single α or using ESTATICS (Figures 7 and 8) regardless of which α‐pairs were used (Figure 8 and Supporting Information Figure S4). Any quantitative imaging protocol comprised of multi‐echo VFA data, such as the MPM protocol used here,^27^, ^28^ can capitalize on these benefits without time penalty, or even use this approach retrospectively with existing datasets. However, $\hat{R_{2}^{*}}$ was accompanied by moderately lower cross‐session repeatability, particularly when compared to the ESTATICS approach (Figure 6). This noise enhancement is likely because of the higher number of model parameters, but can be reduced by including additional flip angles (Figure 2).

In simulations, $\hat{R_{2}^{*}}$ scaled linearly with MWF, but was largely insensitive to residency time. It should be noted that in vivo, even if there is no myelin water compartment, $\hat{R_{2}^{*}}$ will vary spatially because of microstructural susceptibility differences (e.g., because of iron content).^44^ $\hat{R_{2}^{*}}$ sensitivity to MWF was assessed in vivo in the corpus callosum by using the MT saturation (MTsat) measurements of the MPM protocol as a proxy given the common dependence of both MTsat and MWF on myelin volume fraction^45^ (Supporting Information Figure S5). Within the comparatively homogeneous region of interest defined by the corpus callosum, the relationship between $\hat{R_{2}^{*}}$ and MTsat closely matched the MWF dependence predicted by simulations. In particular, there was a monotonic, approximately linear, increase in $\hat{R_{2}^{*}}$ as MTsat increased. Unlike in simulation, the dependence tended to plateau at high MTsat, which may be because of residual tissue heterogeneity not included in our simulations, or be introduced by the use of MTsat as a proxy for MWF.

The spatial variability of the flip angle dependent component $\left(\frac{dR_{2}^{*}}{d\alpha }\right)$ will be specifically driven by spatial variability in myelin water characteristics. This component of $R_{2}^{*}$ had substantial sensitivity to both MWF and residency time. As would be expected, the flip angle dependence manifested more clearly (larger $\frac{dR_{2}^{*}}{d\alpha }$) when the MWF was large and exchange was slow. Simulations showed that $\frac{dR_{2}^{*}}{d\alpha }$ depends approximately quadratically on MWF (Figure 3). The dependence of $\frac{dR_{2}^{*}}{d\alpha }$ on our in vivo MWF proxy (MTsat) was broadly consistent with this (Supporting Information Figure S5).

A heuristic second order model^46^ in MWF can describe this dependence:

(9) $$\frac{dR_{2}^{*}}{d\alpha }=\beta_{1}\mathrm{MWF}+\beta_{2}{\mathrm{MWF}}^{2}.$$

If $\beta_{1}\;$ and $\beta_{2}$ were known, the second order model could be inverted to estimate MWF from the $\frac{dR_{2}^{*}}{d\alpha }$ component measured in vivo. The possibility to estimate MWF in vivo via a simple VFA acquisition would likely be of great interest because it could in principle be applied retrospectively to existing datasets, if sufficiently noise‐robust. Such a simple approach to MWF estimation would overcome limitations of existing approaches, such as extended acquisition time, low spatial resolution or the need for multi‐modal data.^16^, ^32^, ^47^ This intriguing possibility will be investigated further in future work.

### 4.1 Limitations

A highly simplified tissue model with only two compartments, myelin and non‐myelin water, was used to simulate the SPGR signal. The non‐myelin water compartments merged the intra‐ and extra‐cellular water such that compartment‐specific frequency offsets caused by the myelin sheath were also neglected in the simulations and only characterized experimentally via the dependence on WM fiber orientation that results. Dephasing because of other macro‐ or microscopic field inhomogeneities was also ignored. Yet, even in this comparatively simple model, multiple fixed parameters had to be assumed, specifically the longitudinal and transverse relaxation rates of both the myelin and non‐myelin water compartments.

MT effects are prominent in the human brain,^48^ particularly in WM, but have not been included in these simulations. Although this is in keeping with previous works,^16^, ^49^, ^50^ further insights may be delivered by extending the EPG‐X framework to account for this effect by modeling 3 exchanging pools. In addition, it has recently been shown that the excitation can be modified to minimize the effect empirically.^51^ Despite this confound, high levels of agreement were observed between our empirical measurements and our predictions based on two pool modeling.

It has been shown that combining micro‐structural information, such as fiber orientation from diffusion tensor imaging, with multi‐compartment relaxometry modeling can improve the description of the net signal and permit estimation of the MWF.^16^ The gains in model accuracy and robustness come at the cost of longer acquisition times, which are required to collect the data necessary to reduce the degrees of freedom of the model and avoid overfitting. Here, we instead aimed to characterize the impact that the true multi‐compartment nature of tissue has on simpler single compartment $R_{2}^{*}$ estimates, which are typical of neuroscientific studies that sacrifice model complexity to maintain feasible acquisition times.

## 5 CONCLUSION

In this work, a two compartment model was used to investigate the impact of tissue microstructure on single compartment $R_{2}^{*}$ relaxation rate estimates, with particular focus on the flip angle dependence that this produces. Simulations showed good agreement with in vivo data illustrating that MWF and residency time both dictate the observed $R_{2}^{*}$. The heuristic linear model that we propose in this work can partition the α‐dependent and α‐independent components of the single compartment $R_{2}^{*}$. Ideally, the true multi‐compartment nature of the tissue would be faithfully characterized, but if the data to support more advanced modeling are not available, the flip angle independent component may provide a more robust measure owing to its reduced sensitivity to confounding factors such as α and fiber orientation. It may, therefore, be a useful means of reducing spurious variance particularly in multisite studies.

## Supporting information


**Table S1.** Summary of parameters used in the simulations of the net SPGR signal.
**Table S2.** Summary of the data acquired in each of the in vivo sessions.
**Table S3.** Across sessions bias ± CI values for WM and GM for participant 1.
**Figure S1.** Schematic of the per flip angle and ESTATICS methods used to estimate $R_{2}^{*}$. Two signals acquired with different flip angles are represented. Per‐α estimation: $R_{2}^{*}$ is estimated via a log‐linear fit across echo times for each flip angle decay. ESTATICS: a single $R_{2}^{*}$ per flip angle pair is estimated assuming common T_2_* decay across flip angles.
**Figure S2.** effect of fiber orientation with respect to B_0_ on R_2_* estimates for different simulated flip angles. R_2_* recomputed from the linear model of R_2_* dependence on flip angle (dashed lines) approximates the simulated data well (solid lines with diamonds). $\hat{R_{2}^{*}}$ (solid turquoise line with circles) shows the least dependence on fiber orientation θ.
**Figure S3.** $R_{2}^{*}$ computed via simulations for different residency time, MWF and flip angles for a fiber orientation with respect to B_0_ of 90°. (A) Increasing $R_{2}^{*}$ as function of α is observed. (B) A linear model of flip angle dependence fit the simulated data well as evidenced by model errors <2 s^−1^.
**Figure S4.** R_2_* estimated with different flip angle pairs in different sessions. An increase in R_2_* is observed as a function of fiber orientation, θ, especially for high flip angle. $\hat{R_{2}^{*}}$ also increased with θ however it was robust across different flip angle pairs.
**Figure S5.** Dependence of $\hat{R_{2}^{*}}\left(r\right)$ and $\frac{dR_{2}^{*}}{d\alpha }\left(r\right)$ on MTsat measurements in the corpus callosum for 3 participants. MTsat is taken as a proxy for MWF to test the model predictions in vivo given that both metrics depend on the myelin volume fraction. To minimize confounding factors, the model predictions were assessed in the corpus callosum, a comparatively homogeneous ROI (e.g., in terms of iron content or other field perturbers) that has consistent fiber orientation (∼90° with respect to B_0_). This fiber tract was defined by the intersection of the “JHU_MNI_1mm” template^52^ warped to native space and the participant‐specific WM mask defined as those voxels with a WM probability >0.9. The MTsat values were segregated into bins containing 200 voxels to ensure reliable summary statistics. For each bin, mean $\hat{R_{2}^{*}}\left(r\right)$ and $\frac{dR_{2}^{*}}{d\alpha }\left(r\right)$ were calculated and plotted against mean MTsat(r). This was repeated for each of the 3 participants. Both components of $R_{2}^{*}$ increased monotonically with MTsat values and tended to plateau or decrease at higher values. Plateauing and decreasing was predicted for the flip angle dependent component (approximately quadratic dependence on MWF), but was not expected for the flip angle independent component. This may reflect some residual spatial variability in drivers of transverse relaxation or be driven by the use of MTsat as a proxy for MWF.
