# Supplementary material for: Mitigating the impact of flip angle and orientation dependence in single compartment R2* estimates via 2‐pool modeling
Source: Magn Reson Med. 2022 Sep 26;89(1):128–43. doi: 10.1002/mrm.29428 (PMC9827921; doi:10.1002/mrm.29428)
Supplement: Supplementary file 1 — Table S1. Summary of parameters used in the simulations of the net SPGR signal. Table S2. Summary of the data acquired in each of the in vivo sessions. Table S3. Across sessions bias ± CI values for WM and GM for participant 1. Figure S1. Schematic of the per flip angle and ESTATICS methods used to estimate R2*. Two signals acquired with different flip angles are represented. Per‐α estimation: R2* is estimated via a log‐linear fit across echo times for each flip angle decay. ESTATICS: a single R2* per flip angle pair is estimated assuming common T2* decay across flip angles. Figure S2. effect of fiber orientation with respect to B0 on R2* estimates for different simulated flip angles. R2* recomputed from the linear model of R2* dependence on flip angle (dashed lines) approximates the simulated data well (solid lines with diamonds). R2*^ (solid turquoise line with circles) shows the least dependence on fiber orientation θ. Figure S3. R2* computed via simulations for different residency time, MWF and flip angles for a fiber orientation with respect to B0 of 90°. (A) Increasing R2* as function of α is observed. (B) A linear model of flip angle dependence fit the simulated data well as evidenced by model errors <2 s−1. Figure S4. R2* estimated with different flip angle pairs in different sessions. An increase in R2* is observed as a function of fiber orientation, θ, especially for high flip angle. R2*^ also increased with θ however it was robust across different flip angle pairs. Figure S5. Dependence of R2*^(r) and dR2*dα(r) on MTsat measurements in the corpus callosum for 3 participants. MTsat is taken as a proxy for MWF to test the model predictions in vivo given that both metrics depend on the myelin volume fraction. To minimize confounding factors, the model predictions were assessed in the corpus callosum, a comparatively homogeneous ROI (e.g., in terms of iron content or other field perturbers) that has consistent fiber orientation (∼90° with respect to B0). This [file MRM-89-128-s001.docx]

**Supporting Information Figures and Tables**

| **Sequence Parameters** | | **Tissue Parameters** | |
| --- | --- | --- | --- |
| **α [deg]** | 6, 9, 12, 15, 19, 26,  31, 36, 42 | $\mathbf{T}_{\mathbf{1,MW}}$ **[ms]** | 280 |
| **TE_1_:ΔT:TE_6_ [ms]** | 2.56:2.38:14.46 | $\mathbf{T}_{\mathbf{2,}\mathbf{MW}}$**=**$\mathbf{T}_{\mathbf{2,MW}}^{\mathbf{*}}$ **[ms]** | 8 |
| **TR [ms]** | 19.5 | $\mathbf{T}_{\mathbf{1,IE}}$ **[ms]** | 1450 |
| **Spoiler gradient moment** | 6$\pi$ | $\mathbf{T}_{\mathbf{2,IE}}$**=**$\mathbf{T}_{\mathbf{2,IE}}^{\mathbf{*}}$ **[ms]** | 36 |
| **RF spoiling increment** | 144˚ | **MWF** | 0.02:0.02:0.20 |
|  |  | **Residency time [ms]** | 100:100:500 |

*Supporting Information Table 1 – Summary of parameters used in the simulations of the net SPGR signal.*

|  | **Date** | **Field strength** | **Sequence** | **α [deg]** | **TE_1_:ΔT:TE_6_ [ms]** | **TR [ms]** | **b-values**  **[ms]** |
| --- | --- | --- | --- | --- | --- | --- | --- |
| **Participant 1** | 11/02/2020  Session 1 | 7T | SPGR | 6, 9, 26, 42 | 2.56:2.38:14.46 | 19.5 | - |
|  | 10/02/2021  Session 2 | 7T | SPGR | 6, 9, 26, 42 | 2.56:2.38:14.46 | 19.5 | - |
|  | 17/02/2021  Session 3 | 7T | SPGR | 6, 9, 12, 15, 19, 26,  31, 36, 42 | 2.56:2.38:14.46 | 19.5 | - |
|  | 26/05/2021  Session 4 | 3T | Multiband EPI | 88 | 60 | 3320 | 0, 500, 1000, 2300 |
| **Participant 2** | 02/06/2021  Session 5 | 7T | SPGR | 6, 9, 12, 15, 19, 26,  31, 36, 42 | 2.56:2.38:14.46 | 19.5 | - |
| **Participant 3** | 25/05/2022  Session 6 | 7T | SPGR | 6, 9, 26, 42 | 2.56:2.38:14.46 | 19.5 | - |

*Supporting Information Table 2 – Summary of the data acquired in each of the in vivo sessions.*

|  |  | $\mathbf{R}_{\mathbf{2}}^{\mathbf{*}}$ | $\mathbf{R}_{\mathbf{2}}^{\mathbf{*}}$ **ESTATICS** | $\hat{\mathbf{R}_{\mathbf{2}}^{\mathbf{*}}}$ |
| --- | --- | --- | --- | --- |
| Inter Session  bias±CI [1/s] | GM | -0.41±18.48 | 0.07±13.79 | -0.31±20.26 |
|  | WM | -0.75±8.61 | 0. 23±6.08 | 0.21±9.44 |

*Supporting Information Table 3* – *Across sessions bias±CI values for WM and GM for participant 1.*


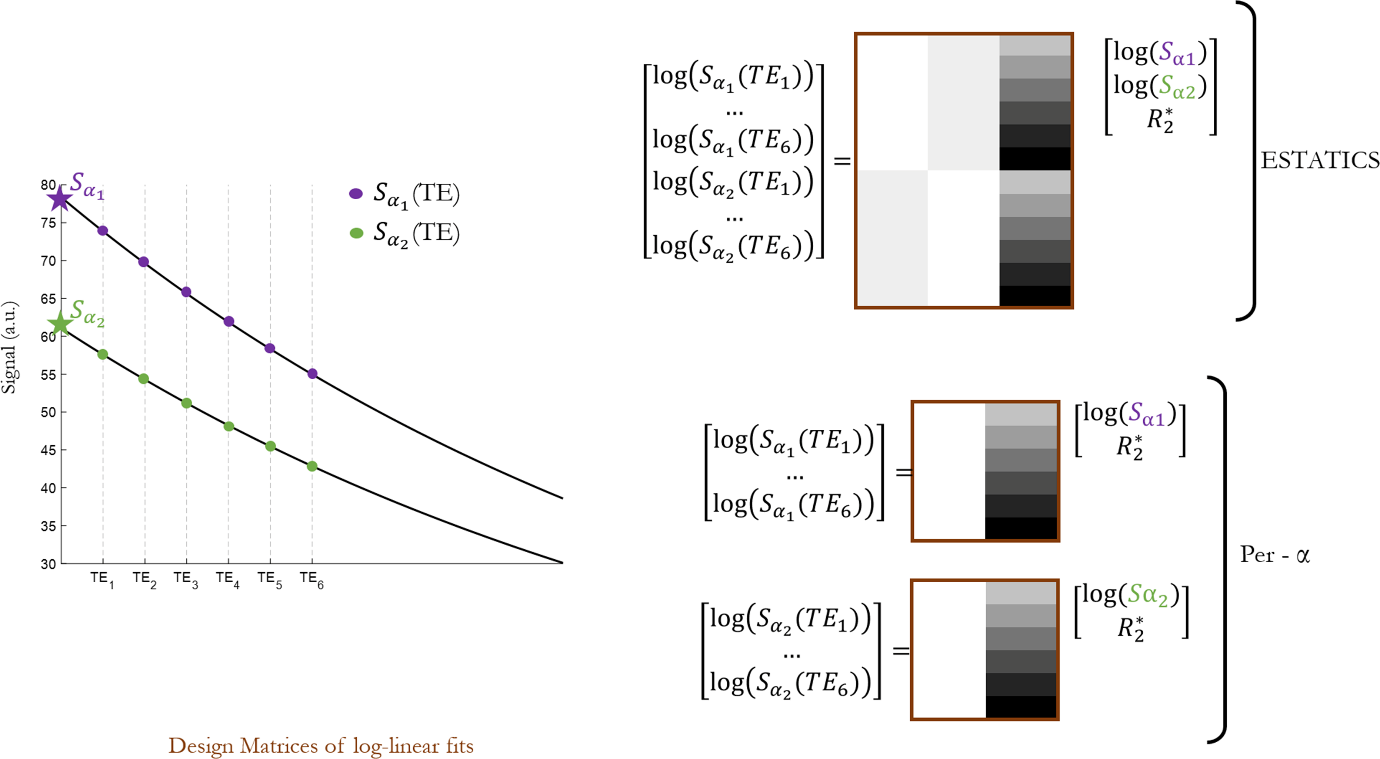


*Supporting Information Figure S1 – Schematic of the per flip angle and ESTATICS methods used to estimate* $R_{2}^{*}$*. Two signals acquired with different flip angles are represented. Per-*$\alpha$ *estimation:* $R_{2}^{*}$ *is estimated via a log-linear fit across echo times for each flip angle decay. ESTATICS: a single* $R_{2}^{*}$ *per flip angle pair is estimated assuming common T_2_* decay across flip angles.*


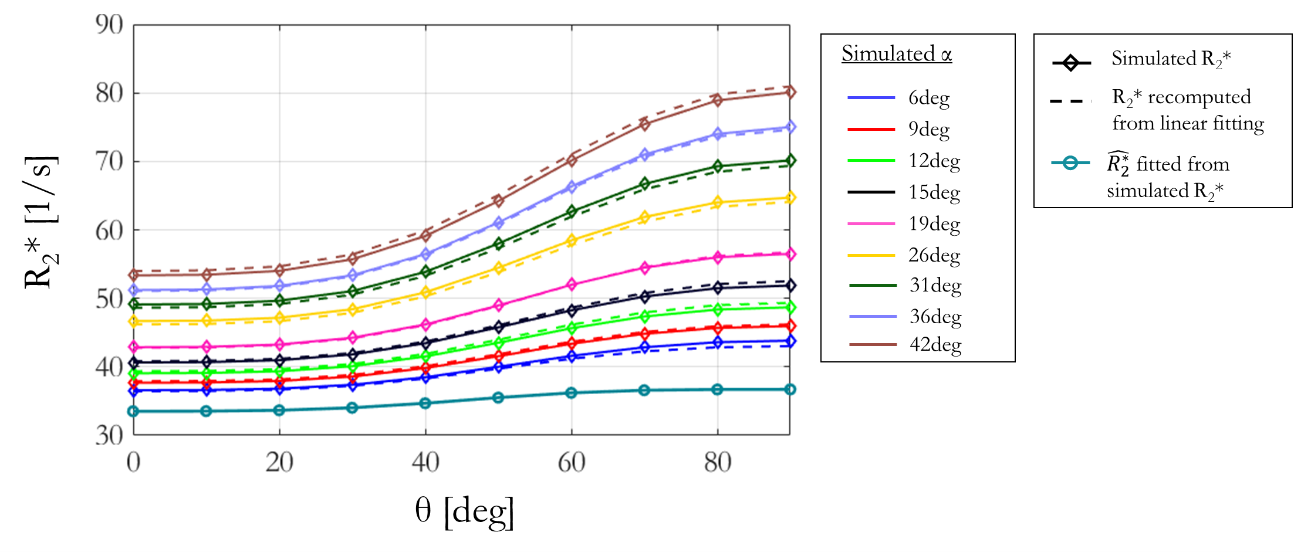


*Supporting Information Figure S2 – effect of fibre orientation with respect to B_0_ on R_2_* estimates for different simulated flip angles. R_2_* recomputed from the linear model of R_2_* dependence on flip angle (dashed lines) approximates the simulated data well (solid lines with diamonds).* $\hat{R_{2}^{*}}$ *(solid turquoise line with circles) shows the least dependence on fibre orientation* θ*.*


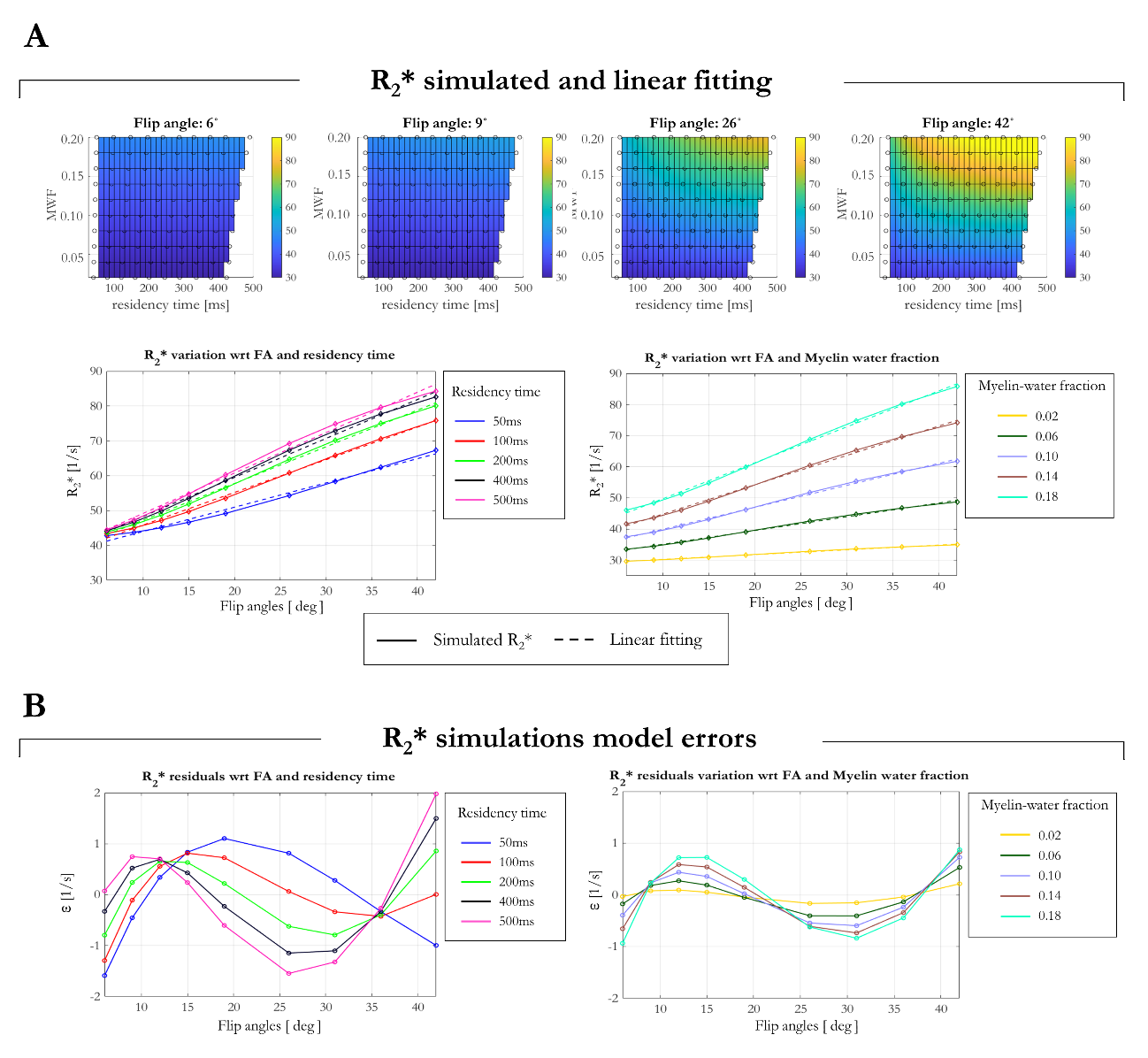


*Supporting Information Figure S3 –* $R_{2}^{*}$ *computed via simulations for different residency time, MWF and flip angles for a fibre orientation with respect to B_0_ of 90˚. A) Increasing* $R_{2}^{*}$ *as function of α is observed. B) A linear model of flip angle dependence fit the simulated data well as evidenced by model errors < 2s^-1^.*


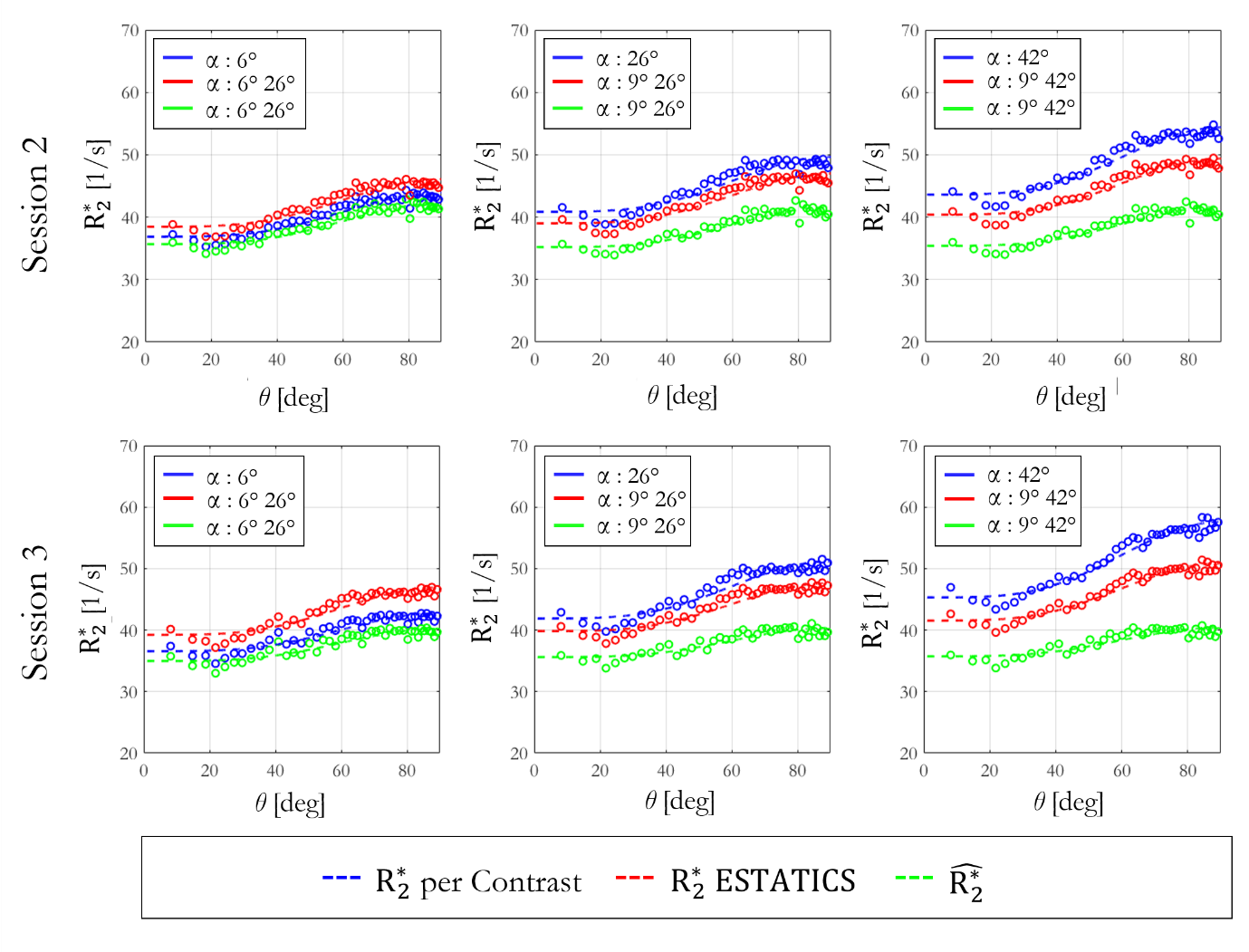


*Supporting Information Figure S4 – R_2_* estimated with different flip angle pairs in different sessions. An increase in R_2_* is observed as a function of fibre orientation,* θ*, especially for high flip angle.* $\hat{R_{2}^{*}}$ *also increased with* θ *however it was robust across different flip angle pairs*.


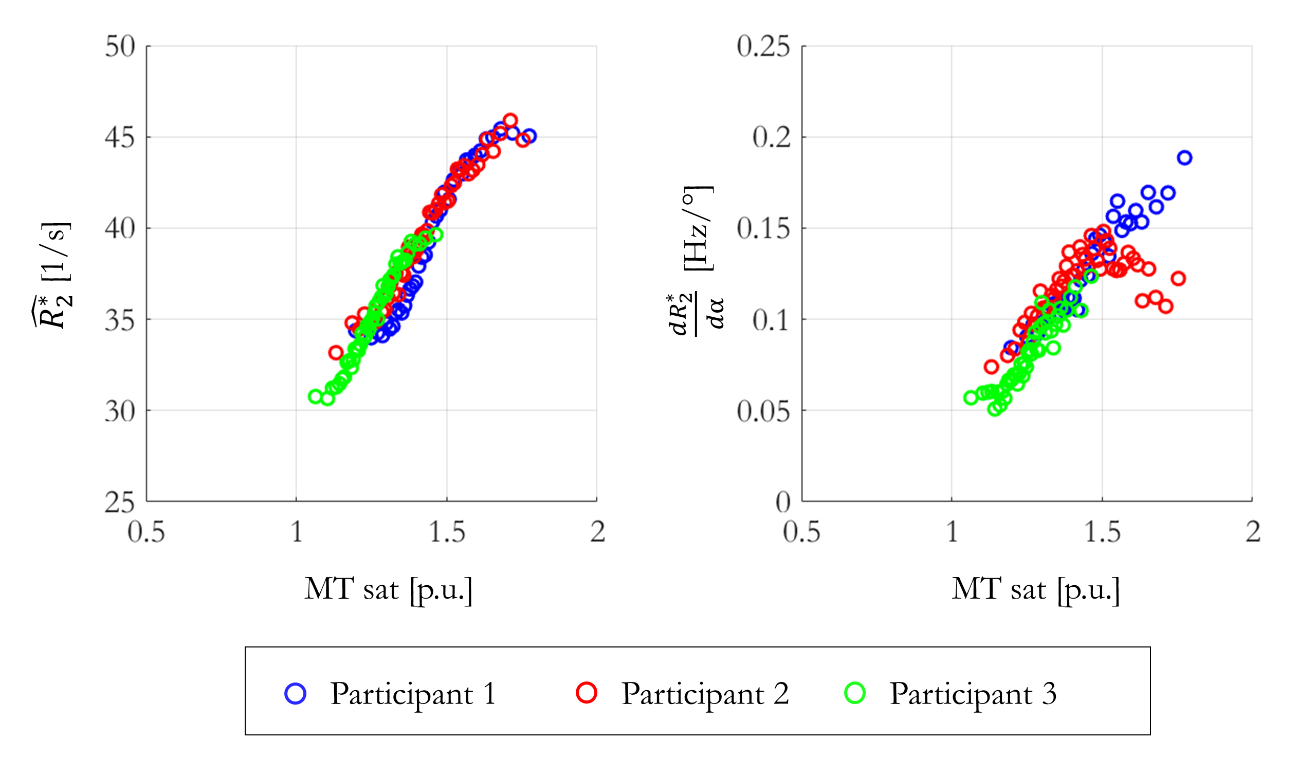


*Supporting Information Figure S5 –* *dependence of* $\hat{R_{2}^{*}}(r)$ *and* $\frac{dR_{2}^{*}}{d\alpha}(r)$ *on MTsat measurements in the corpus callosum for three participants. MTsat is taken as a proxy for MWF to test the model predictions in vivo given that both metrics depend on the myelin volume fraction. To minimise confounding factors, the model predictions were assessed in the corpus callosum, a comparatively homogeneous ROI (e.g. in terms of iron content or other field perturbers) that has consistent fibre orientation (approximately 90˚ with respect to B_0_). This fibre tract was defined by the intersection of the ‘JHU_MNI_1mm’ template* [52] *warped to native space and the participant-specific WM mask defined as those voxels with a WM probability > 0.9. The* $MTsat$ *values were segregated into bins containing 200 voxels to ensure reliable summary statistics. For each bin, mean* $\hat{R_{2}^{*}}(r)$ *and* $\frac{dR_{2}^{*}}{d\alpha}(r)$ *were calculated and plotted against mean* $MTsat(r)$*. This was repeated for each of the three participants. Both components of* $R_{2}^{*}$ *increased monotonically with MTsat values and tended to plateau or decrease at higher values. Plateauing and decreasing was predicted for the flip angle dependent component (approximately quadratic dependence on MWF), but was not expected for the flip angle independent component. This may reflect some residual spatial variability in drivers of transverse relaxation or be driven by the use of MTsat as a proxy for MWF.*
